# The impact of multiple sclerosis on wellbeing, productivity, and societal relations

**DOI:** 10.1055/s-0045-1809400

**Published:** 2025-06-20

**Authors:** Vinicius Eduardo Vergani, Bruna Rocha Silveira, Gustavo San Martin Elexpe Cardoso, Jean Faber, Denis Bernardi Bichuetti

**Affiliations:** 1 Universidade Federal de São Paulo, Escola Paulista de Medicina, São Paulo SP, Brazil.; 2 Amigos Múltiplos pela Esclerose, Guarulhos SP, Brazil.; 3 Universidade Federal de São Paulo, Escola Paulista de Medicina, Disciplina de Neurociências, São Paulo SP, Brazil.; 4 Universidade Federal de São Paulo, Escola Paulista de Medicina, Disciplina de Neurologia, São Paulo SP, Brazil.

**Keywords:** Multiple Sclerosis, Employment, Insurance, Disability, Social Impact Indicators, Disability Studies

## Abstract

**Background:**

Multiple sclerosis (MS) is the main cause of nontraumatic neurological disabilities in the population under 50 years of age.

**Objective:**

To evaluate the most prevalent symptoms in a national sample of people with MS and to analyze their correlation with disease characteristics, demographics, quality of life, employment status, and use of social benefits.

**Methods:**

Cross-sectional, online, self-reported survey, concerning demographic and clinical data, employment status, and use of social benefits.

**Results:**

A total of 466 patients answered the survey. The median age at onset was 30 years, the current age, of 39, and disease duration was 8 years. Furthermore, the median patient determined disease steps (PDDS) was 2, which indicates minor to moderate disability. The median MS impact and walking scale scores were 31 and 20%, which denotes minor to moderate quality of life and mobility compromise. Among the participants, 43% suffered fatigue and 51% reported not sleeping well. Unemployed patients had delayed diagnosis and higher disability rates. Furthermore, half of the unemployed patients are receiving some social benefit, compared with only 6.5% of the employed patients.

**Conclusion:**

The current study presents symptom prevalence in a national sample of patients with MS and discloses that those with a diagnosis delay and more disability have higher rates of unemployment and use of social benefits. Strategies for earlier diagnosis and better treatment plans can not only reduce patient disability but, possibly, increase employment retention and reduce the use of social benefits.

## INTRODUCTION

Multiple sclerosis (MS) is a chronic inflammatory and demyelinating disease that affects the central nervous system. 1 People with MS (PwMS) suffer from cumulative neurological disabilities and 60% of them are unable to walk without assistance 20 years after the first symptom if untreated. 2 3 This disease debuts between 20 and 40 years of age, the peak of modern productive life, thus compromising quality of life (QoL) and efficiency, negatively impacting personal autonomy, social relations, employment status, and remuneration. 4

MS is a treatable disease and is widely known that with earlier diagnosis and treatment, accumulation of disability is postponed or may even never happen. 2 5 Furthermore, it has been proved that early treatment can indeed be economically healthy, as the disease's cost increases with rising disabilities, mostly due to indirect costs related to loss of production and use of social benefits, in association with treatment for neurological sequelae. 6 7 As it strikes early, MS is known to be the leading cause for neurological disabilities under the age of 50 years worldwide. 8

We have previously shown that 40% of PwMS in Brazil are unemployed earlier in life, even those with high educational status. This rate also increases with disease duration. 9 Although there are many real life, prospective, and retrospective national studies reporting the benefits of early and personalized therapy in reducing future disability for MS in Brazil, 10 the factors that influence employment retention, associated symptoms, and QoL in this population are vastly underreported.

Intending to understand the major symptoms of Brazilian PwMS and their relation to clinical data, QoL, employment status, and use of social benefits, we performed a national survey. Furthermore, we aimed to identify the key factors that contribute to disability accumulation, personal suffering, and social costs for this cohort.

## METHODS

This was a cross-sectional, online, self-reported survey, performed from March to July 2022. This study was approved by Universidade Federal de São Paulo's Ethics Committee, under the CAAE 54011121.3.0000.5505 and register 5.243.398, in February 2022. All patients signed an online consent form. The survey was spread by the patient-committed organization Amigos Múltiplos pela Esclerose (AME, http://www.amigosmultiplos.org.br/ ) by e-mail and social media announcement, as previously performed by our group. 9

The survey's first part included questions on social, demographic, educational status, employment status, social benefits, age of disease onset, and age of diagnosis, intending to draw the profile of this population like previously published. 9 For practical purposes, in this manuscript, we defined “employed” as anyone with a regular income by Brazilian standards, be it under a specific company or third party, business owner or self-employed. By consequence, the designation “unemployed” meant someone with no regular income or that does not take part in any productivity work. The term social benefit was used to define any financial governmental aid, such as disease related retirement, temporary leave aid/insurance, temporary or permanent disability aid, *bolsa família* (a national money redistribution program), or any other social program that generates income.

The survey progressed to clinical evaluation with already validated questionnaires. The first is the Patient Determined Disease Scale (PDDS) for clinical disability, rated 1 to 9. In which (1) normal: may have some mild symptoms but they do not limit activity; (2) mild disability have some symptoms but they are minor and have a small effect on lifestyle; (3) moderate disability: no walking limitation, but significant problems due to MS that limit daily activities in other ways; (4) gait disability: walking impairment without the help of a cane; (5) early cane: walking impairment with the use of a cane for most part of the day; (6) late cane: cane or crutch is needed to walk 25 feet, may use a scooter or wheelchair for greater distances; (7) bilateral support is needed to walk 25 feet, may use a scooter or wheelchair for greater distances; (8) wheelchair or scooter dependance; and (9) bedridden. 11

The second is the Brazilian Multiple Sclerosis Impact Scale – 29 (MSIS-29-BR: 0–116) for QoL, where higher scores correspond to higher disability and QoL compromise. 12 Followed by the Brazilian Multiple Sclerosis Walking Scale – 12 (MSWS-12-BR) for walking impairment (12–60), in which higher scores correspond to higher walking impairment. 13 The Fatigue Severity Scale (FSS), regarding MS related fatigue (9–63), with higher scores corresponding to higher MS related fatigue. 14 The Brief Pain Inventory (BPI: 0–10), with higher scores corresponding to more severe pain. 15 And the Insomnia Severity Index (ISI) for sleep quality, which uses Yes/No questions and a 0 to 4 scale (0 = normal and 4 = severe insomnia). 16

The Excel (Microsoft Corp.) software was used to tabulate the data and GraphPad Prism 10.0.2 (GraphPad Software, Inc.) and MATLAB R2018a (MathWorks Inc.) were used for the statistical analysis.

The D'Agostino-Pearson and Kolmogorov-Smirnov tests were used to evaluate the departure from normality and data are presented as mean and standard deviation (SD) if met normal distribution criteria and median and first to third quartiles if not. We further decided to carry out only pairwise statistical analyses to better understand the relationships between variables. We applied an unpaired *t* -test for all comparisons and for those samples without normal origin. Also, we confirmed significances with Mann-Whitney test. The chi-squared or Fisher's exact tests were used when appropriate.

For a better visualization, all results are presented by means ± standard errors (  ), except for the ‘Occupation’ and ‘Benefits’ variables that were presented as percentages  with its 95%CIs:  .

All correlations were evaluated through linear regressions with Pearson's coefficients, tested by the *t* -test. Correlations between ‘PDDS x Disease duration’ and ‘PDDS x Diagnosis delay’ were performed using log of their histograms (with 8 bins). For all analysis the significance level was set at *p* < 0.05.

## RESULTS

We obtained 467 answers from all five Brazilian geopolitical regions (North, Northeast, Central-West, Southeast, and South). One patient was excluded because answers were sent by email and not within the proposed platform, thus, 466 answers were included for analysis. No duplicated entries were found. Considering that the estimated number of people living with MS in Brazil is around 40 thousand to 60 thousand, 17 18 this sample comprises approximately 1% of PwMS in the country.

This sample represents a young population, with median (first–third quartiles) age at MS onset of 30.0 (23.0–36.0), current age of 39.0 (32.0–47.8), diagnosis delay of 1.0 (0.0–3.0), and disease duration of 8.0 (4.0–15.0), all being analyzed in years, as shown in Table 1 .

**Table 1:** Clinical and demographic data

			466 patients: n (%)	Median (1st–3rd quartiles; minimum–maximum)
Gender	Male		66 (14.2%)	
	Female		400 (85.8%)	
Ethnicity	Caucasian		341 (7.2%)	
	Of African-descent		24 (5.2%)	
	Brown		95 (20.4%)	
	Asian		5 (1.1%)	
	Indigenous		1 (0.1%)	
Region of birth	North		3 (0.6%)	
	Northeast		57 (12.2%)	
	Center-West		20 (4.3%)	
	South		65 (14%)	
	Southeast		321 (68.9%)	
Current region of residence	North		2 (0.4%)	
	Northeast		51 (10.9%)	
	Center-West		24 (5.2%)	
	South		68 (14.6%)	
	Southeast		321 (68.9%)	
Marital status	Married		274 (58.8%)	
	Divorced		35 (7.5%)	
	Single (never married)		153 (32.8%)	
	Widowed		4 (0.9%)	
Level of education	Elementary school		7 (1.5%)	
	High school		117 (25.1%)	
	University degree		153 (32.8%)	
	Postgraduation		189 (40.6%)	
Employment status	Yes		307 (65.9%)	
	No		159 (34.1%)	
Receiving social benefits	Yes		98 (20.9%)	
	No		368 (79.1%)	
Current age (years)				39.0 (32.0–47.8); (18.0–80.0)
Age at onset (years)				30.0 (23.0–36.0); (5.0–66.0)
Disease duration (years)				8.0 (4.0–15.0); (0.0–60.0)
Diagnosis delay (years)				1.0 (0.0–3.0); (0.0–33.0)
PDDS (1–9)				2.0 (1.0–4.0); (1. –8.0)
MSIS-29-BR	Physical Domain (0– 80)			15.0 (5.0–31.0); (0.0–80.0)
	Psychological Domain (0–36)			16.0 (8.0–24.0); (0.0–36.0)
	Total (0–116)			31.0 (16.0–54.8); (0.0–116.0)
MSWS-12-BR	(12–60)			20.0 (12. –35.8); (12.0–60.0)
FSS (9 to 63)	Yes (>41)		199 (42.7%)	35.0 (18.0–54.0); (9.0–63.0)
	No (<42)		267 (57.3%)	
BPI (Short Form)	Suffered from pain in the last week	Yes, n (%)	221 (47.4%)	
		No, n (%)	245 (52.6%)	
Pain severity (0 to 10)				3.0 (0.0–6.0); (0.0–10.0)
Insomnia	Difficulty falling asleep	Yes, n (%)	228 (49%)	
		No, n (%)	237 (51%)	
	Satisfaction with sleep quality	Yes, n (%)	217 (46.8%)	
		No, n (%)	247 (53.2%)	
ISI	Difficulty falling asleep	0	179 (38.5%)	
		1	78 (16.8%)	
		2	82 (17.6%)	
		3	62 (13.4%)	
		4	64 (13.7%)	
	Difficulty staying asleep	0	156 (33.5%)	
		1	84 (18.1%)	
		2	95 (20.5%)	
		3	64 (13.7%)	
		4	66 (14.2%)	
	Problems waking up too early	0	159 (34.3%)	
		1	68 (14.7%)	
		2	88 (19.0%)	
		3	62 (13.4%)	
		4	86 (18.6%)	

Abbreviations: BPI, brief pain inventory; FSS, fatigue severity scale; ISI, insomnia severity index; MSIS-29-BR, multiple sclerosis impact scale–29–BR; MSWS-12-BR, multiple sclerosis walking scale-12-BR; PDDS, patient determined disease steps.

Clinical disability measured by the PDDS was mild to moderate (median: 2.0; 1.0–4.0), and the majority had been living with MS for a median of 8 years ( Table 1 ). There was a high range of disease duration (0–60 years), which represents a population from a large time span, with access to diagnostic tools and treatments in different moments of history, with potential bias in the disability analysis.

The therapeutic scenario for MS in Brazil can be divided in two periods, before and after 2012. This year marks the availability of natalizumab as the first highly efficacious therapy in the Brazilian Unified Health System (Sistema Único de Saúde, SUS, in Portuguese), responsible for providing treatment for most PwMS. Other highly efficacious drugs were only made available later: fingolimod in 2015 and alemtuzumab in 2022. Assuming that PwMS that were diagnosed and treated prior to 2012 did not have access to highly efficacious therapies, we further divided this sample into those living with MS for more or less than 10 years. These groups were compared in search of a possible timestamp that represents the access to more efficacious therapies and, thus, a change in treatments standards ( Table 2 ).

**Table 2:** Clinical and demographic data according to living with MS for more or less than 10 years

Category		Disease duration < 10 years (n = 283)	Disease duration > 10 years (n = 183)	*p* -value
Age at onset (years)		31.0 (25.0–37.0); (12.0–66.0)	27.0 (21.0–34.0); (5.0–64.0)	< 0.05
Age at diagnosis (years)		32.0 (26.0–39.0); (12.0–66.0)	31.0 (25.0–40.0); (9.0–71.0)	
Diagnosis delay (years)*		1.0 (0.0–1.5); (0.0–9.0)	2.0 (1.0–7.0); (0.0–33.0)	< 0.05
Current age (years)		36.0 (30.5–42.0); (18.0–68.0)	45.0 (39.0–55.5); (24.0–80.0)	< 0.05
Disease duration (years)		5.0 (3.0–7.0); (0.0–10.0)	16.0 (13.0–22.0); (11.0–60.0)	< 0.05
Time of diagnosis		3.0 (1.5–6.0); (0.0–10.0)	13.0 (9.0–18.0); (0.0–57.0)	< 0.05
Employment status	Employed	209 (73.8%)	98 (53.5%)	< 0.01
	Unemployed	74 (26.2%)	85 (46.5%)	
BPI (short form)		3.0 (0.0–6.0); (0.0–10.0)	5.0 (1.0–7.0); (0.0–10.0)	0.02
	Yes	121 (42.8%)	100 (54.6%)	
	No	162 (57.2%)	83 (45.4%)	
Fatigue (0–63)		31.0 (15.5–51.0); (9.0–63.0)	44.0 (22.0–56.0); (9.0–63.0)	< 0.05
	Yes	105 (37.1%)	94 (51.4%)	
	No	178 (62.9%)	89 (48.6%)	
MSIS-29-BR	Physical domain (0–80)	10.0 (3.0–24.5); (0.0–72.0)	24.0 (9.0–42.0); (0.0–80.0)	< 0.05
	Psychological domain (0–36)	15.0 (8.0–24.0); (0.0–36.0)	17.0 (8.0–25.0); (0.0–36.0)	0.40
	Total (0–116)	27.0 (13.5–45.5); (0.0–107.0)	39.0 [(20.0–65.5); (0.0–116.0)]	< 0.05
MSWS-12-BR (12–60)		15.0 (12.0–28.5); (12.0–59.0)	27.0 (16.5–42.0); (12.0–60.0)	<0.05
PDDS (1–9)		1.0 (1.0–2.0); (1.0–8.0)	3.0 (1.0–5.0); (1.0–8.0)	< 0.05

Abbreviations: BPI, brief pain inventory; MSIS-29-BR, Multiple Sclerosis Impact Scale–29–BR; MSWS-12-BR, Multiple Sclerosis Walking Scale-12-BR; PDDS, patient determined disease steps. Note: Data expressed as n (%) or median (1st–3rd quartiles); (minimum–maximum), when applicable.

The comparison of the employed and unemployed population disclosed that PwMS that retain an occupation, with the same age at disease onset, were diagnosed earlier and have milder PDDS and symptoms as evaluated by all the clinical scales ( Table 3 ). When splitting the sample by those that developed MS prior to and after 2012, it became clearer that diagnosis delay was longer for those living with MS for more than 10 years, possibly reflecting medical knowledge and diagnostic criteria at the time, allied to improvement in diagnosis dissemination and healthcare over the years. Obviously, those with longer disease duration also scored worse in all QoL measures and disability indexes, as measured by the PDDS ( Table 2 ).

**Table 3:** Demographic and clinical data according to employment status

Category		Employed (n = 307)	Unemployed (n = 159)	*p* -value
Age at onset (years)		30.0 (23.0–35.0); (5.0–64.0)	30.0 (23.0–38.8); (8.0–66.0)	0.21
Age at diagnosis (years)		31.0 (25.0–37.3); (9.0–71.0)	34.0 (27.0–41.0); (13.0–66.0)	0.03
Diagnosis delay (years)		1.0 (0.0–3.0); (0.0–30.0)	1.0 (0.0–3.0); (0.0–33.0)	0.02
Current age (years)		38.0 (31.8–45.0); (18.0–80.0)	43.5 (36.0–55.8); (18.0–80.0)	< 0.05
Disease duration (> 10 years)		7.0 (4.0–12.0); (0.0–54.0)	11.0 (6.0–17.0); (0.0–60.0)	< 0.05
	Yes	98 (31.9%)	85 (53.5%)	< 0.05
	No	209 (68.1%)	74 (46.5%)	
Receiving social benefits		307	159	< 0.01
	Yes	20 (6.5%)	77 (48.4%)	
	No	287 (93.5%)	82 (51.6%)	
BPI (short form)		3.0 (0.0–6.0); (0.0–10.0)	5.0 (1.0–7.0); (0.0–10.0)	< 0.05
	Yes	135 (44%)	86 (54%)	
	No	172 (56%)	73 (46%)	
Fatigue (0–63)		31.0 (16.8–50.3); (9.0–63.0)	45.5 [(21.0–58.0); (9.0–63.0)]	< 0.05
	Yes (>41)	112 (36.4%)	87 (54.7%)	
	No (<42)	195 (63.6%)	72 (45.3%)	
MSIS-29-BR	Physical domain (0–80)	9.0 (3.0–22.0); (0.0–69.0)	29.0 [(15.0–48.8); (0.0–80.0)]	< 0.05
	Psychological domain (0–36)	14.0 (7.0–22.3); (0.0–36.0)	18.5 [(10.0–29.0); (0.0–36.0)]	< 0.05
	Total (0–116)	25.0 (13.0–41.0); (0.0–99.0)	46.0 [(27.5–73.3); (0.0–116.0)]	< 0.05
MSWS-12-BR (12–60)		15.0 (12.0–26.0); (12.0–60.0)	34.5 (19.0–47.0); (12.0–60.0)	< 0.05
PDDS (1–9)		1.0 (1.0–2.0); (1.0–8.0)	4.0 (2.0–5.0); (1.0–8.0)	< 0.05

Abbreviations: BPI, brief pain inventory; MSIS-29-BR, multiple sclerosis impact scale–29–BR; MSWS-12-BR, multiple sclerosis walking scale-12-BR; PDDS, patient determined disease steps. Note: Data expressed as n (%) or median (1st–3rd quartiles); (minimum – maximum)], when applicable.

The unemployed population were also older at diagnosis with small, but statistically significant difference, in diagnosis delay, and slightly older at time of survey answer ( Table 3 ). This population also presented higher scores for all the clinical scales, meaning that they had more MS symptoms and neurological impact than the others. Nearly 50% of unemployed PwMS alleged to receive some sort of social benefit, contrasting to only 6.8% of those that are employed.

Those living with MS for more than 10 years were obviously older but also presented longer diagnosis delay and worse scores in all clinical scales ( Table 2 ), possibly reflecting medical knowledge, rigid and escalating clinical protocols, and absence of higher efficacy drugs at time of early diagnosis.

**Figure 1:**
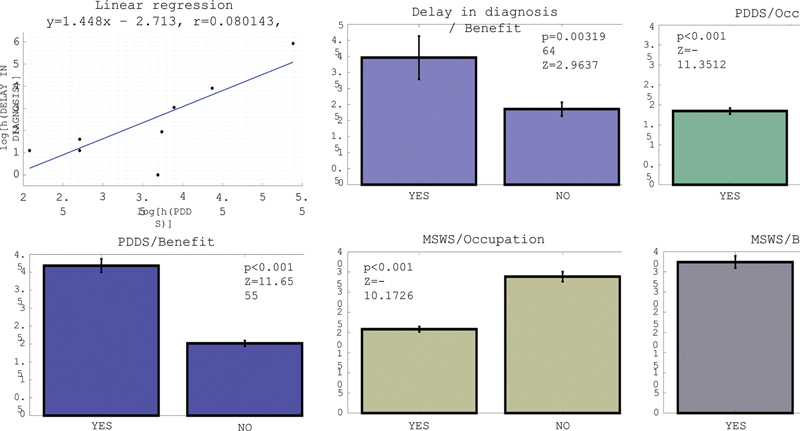
Multivariate analysis of the essential data.

The multivariate analysis disclosed the following relevant results ( Figure 1 ):

- The Federative State of residence did not correlate with diagnosis delay nor clinical disability, suggesting the disease to be uniformly treated nationwide;
- Diagnosis delay correlated with higher clinical disability, unemployment, receiving social benefits, and being divorced;
- Higher clinical disability correlated with having more pain and fatigue and to receiving any social benefit;
- Most clinical variables had stronger correlations with clinical disability over disease duration;
- Being unemployed correlated not only with clinical disability, but having higher fatigue, walking impairment, and pain;
- Living with MS for a longer period correlated with receiving social benefits.

## DISCUSSION

The current work strengthens our prior study 19 with the addition of social and clinical data within a multivariate analysis. This sample of 466 patients represents nearly 1% of the people living with MS from different ethnical and educational backgrounds in all 5 geopolitical regions of Brazil. Once again, the most striking information is that, even within a sample of young PwMS with mild clinical compromise, of whom 72% had academic degrees, only 65% are employed or have a paid occupation, and 21% receive some sort of social benefit, reflecting the social and economic impact of this condition.

Furthermore, clinical disability and MS related symptoms (fatigue, walking impairment, pain and sleep), independently correlated with a longer delay in diagnosis and the odds of being unemployed. This delay in diagnosis and treatment is directly and independently associated with higher disability, distress, unemployment, and possible increase in collective expenses.

Invisible symptoms, such as mild walking impairment, fatigue, pain, insomnia, and visual disturbances, are a great source of discomfort. 20 As these are unnoticeable to the accompanying person, PwMS tend to be labeled as annoyed and lazy, which magnifies the social prejudice and suffering of these patients. Acknowledging what PwMS suffer, and that it is a treatable condition, is of uttermost importance to reduce social prejudice and facilitate inclusion in work, social, and leisure activities, thus directly improving wellbeing and QoL.

This condition is a great example of how modern medicine can influence a disease's natural history and thus its impact not only on one's personal life, but society itself. Treating MS earlier and postponing disability impacts on the annual disease's costs in many countries, including Brazil, 2 6 7 21 and this has been proven in populational studies revealing that PwMS at 40 years of age have half the disability in 2017, compared to those at same age in 1997. 5 The effect of delaying or even halting disability progression, thus changing the disease's natural history, is even stronger if therapy is implemented earlier and with an individualized protocol that considers multiple disease characteristics, rather than a rigid and escalating approach. 2 22 23 24 25

Online surveys are useful for reaching a higher number of patients with a rare disease, but they are not without limitations. Diagnosis certainty was assured by including only patients affiliated with organizations, but we did not have access to individualized clinical data. Participants that are older, with more severe disease and cognitive impairment, might have been uncomfortable in answering an electronic survey. Additionally, this methodology might not reach those in more vulnerable situations, such as illiterate patients, or those with restricted or no internet access. Although this leads to selection bias, we ended up with a young and qualified population that still harbored 35% of unemployment, strengthening the value of this work.

Though individual treatments and direct financial implications were not measured in this study, as this would be a complex task, especially in a country with huge economical divergence, these results can be used to prove the devastating social effect of MS and the consequences of treatment delay. Furthermore, the fact that 21% pledged to receive a social benefit does not mean that they are the only group in need of social support. The country's resources might be limited, and a rare disease may not be considered priority, thus, there might be more patients in vulnerable situations than those actually supported by specific social programs.

In conclusion, this simple survey is in agreement with worldwide data that higher disability impacts PwMS WoL, labor market insertion and productivity. Although indirectly, we can also state that those that have suffered their first symptom after 2012 might have benefited from more efficacious treatments available in Brazil. What we cannot determine from this survey is whether a rigid escalating protocol, as used for many years in Brazil and only recently updated, 19 or a personalized approach where “all therapies should be made available for individualized treatment”, as internationally recommended, 23 24 can have an even higher impact on employment retention, wellbeing, and long-term overall cost reduction. Nevertheless, as with any chronic life-long disease, earlier diagnosis and faster implementation of adequate therapies have proven to alter the disease course. These approaches might also reduce the social impact for the individual and collective.
